# Approximation of the generalized Cauchy–Jensen functional equation in $C^{*}$-algebras

**DOI:** 10.1186/s13660-018-1824-6

**Published:** 2018-09-12

**Authors:** Prondanai Kaskasem, Chakkrid Klin-eam

**Affiliations:** 1 Department of Mathematics, Faculty of Science, Naresuan University, Phitsanulok, Thailand; 2 Research Center for Academic Excellence in Mathematics, Naresuan University, Phitsanulok, Thailand

**Keywords:** 39B52, 47H10, Cauchy–Jensen functional equations, Hyers–Ulam–Rassias stability, $C^{*}$-algebras, Fixed point theorem

## Abstract

In this paper, we prove Hyers–Ulam–Rassias stability of $C^{*}$-algebra homomorphisms for the following generalized Cauchy–Jensen equation:

$$ \alpha\mu f \biggl(\frac{x+y}{\alpha}+z \biggr) = f(\mu x) + f(\mu y) +\alpha f( \mu z), $$

for all $\mu\in\mathbb{S}:= \{ \lambda\in\mathbb{C} \mid|\lambda| =1\}$ and for any fixed positive integer $\alpha\geq2$, which was introduced by Gao et al. [*J. Math. Inequal.* 3:63–77, 2009], on $C^{*}$-algebras by using fixed poind alternative theorem. Moreover, we introduce and investigate Hyers–Ulam–Rassias stability of generalized *θ*-derivation for such functional equations on $C^{*}$-algebras by the same method.

## Introduction and preliminaries

Throughout this paper, let $\mathbb{N}$, $\mathbb{R}$ and $\mathbb{C}$ be the set of natural numbers, the set of real numbers, the set of complex numbers, respectively. The stability problem of functional equations was initiated by Ulam in 1940 [2] arising from concern over the stability of group homomorphisms. This form of asking the question is the object of stability theory. In 1941, Hyers [3] provided a first affirmative partial answer to Ulam’s problem for the case of approximately additive mapping in Banach spaces. In 1978, Rassias [4] gave a generalization of Hyers’ theorem for linear mapping by considering an unbounded Cauchy difference. A generalization of Rassias’ result was developed by Găvruţa [5] in 1994 by replacing the unbounded Cauchy difference by a general control function.

In 2006, Baak [6] investigated the Cauchy–Rassis stability of the following Cauchy–Jensen functional equations:

$$\begin{aligned}& f \biggl(\frac{x+y}{2} + z \biggr) + f \biggl( \frac{x-y}{2}+z \biggr) = f(x) + 2f(z), \\& f \biggl( \frac{x+y}{2} + z \biggr) - f \biggl( \frac{x-y}{2} + z \biggr) = f(y), \end{aligned}$$

or

$$ 2f \biggl( \frac{x+y}{2} + z \biggr) = f(x) + f(y) +2f(z) $$

for all $x,y,z \in X$, in Banach spaces.

The fixed point method was applied to study the stability of functional equations by Baker in 1991 [7] by using the Banach contraction principle. Next, Radu [8] proved a stability of functional equation by the alternative of fixed point which was introduced by Diaz and Margolis [9]. The fixed point method has provided a lot of influence in the development of stability.

In 2008, Park and An [10] proved the Hyers–Ulam–Rassias stability of $C^{*}$-algebra homomorphisms and generalized derivations on $C^{*}$-algebras by using alternative of fixed point theorem for the Cauchy–Jensen functional equation $2f ( \frac{x+y}{2} + z ) = f(x) + f(y) +2f(z)$, which was introduced and investigated by Baak [6]

The definition of the generalized Cauchy–Jensen equation was given by Gao et al.[1] in 2009 as follows.

### Definition 1.1

([1])

Let *G* be an *n*-divisible abelian group where $n \in\mathbb{N}$ (i.e. $a \mapsto na \mid G \to G$ is a surjection) and *X* be a normed space with norm $\|\cdotp\|_{X}$. For a mapping $f : G\to X$, the equation

$$ n f \biggl( \frac{x+y}{n} + z \biggr) = f(x) + f(y) + nf(z) $$

for all $x,y,z \in G$ and for any fixed positive integer $n \geq2$ is said to be a generalized Cauchy–Jensen equation (GCJE, shortly).

In particular, when $n = 2$, it is called a Cauchy–Jensen equation. Moreover, they gave the following useful properties.

### Corollary 1.2

([1])

*For a mapping* $f : G \to X$, *the following statements are equivalent*.

(i) *f* *is additive*.
(ii) $n f (\frac{x+y}{n} + z ) = f(x) + f(y) +nf(z)$, *for all* $x,y,z \in G$.
(iii) $\Vert nf (\frac{x+y}{n} + z ) \Vert _{X} \geq\| f(x) + f(y) + n f(z)\|_{X}$, *for all* $x,y,z \in G$.

It is obvious that a vector space is *n*-divisible abelian group, so Corollary 1.2 works for a vector space *G*.

All over this paper, $\mathbb{A}$ and $\mathbb{B}$ are $C^{*}$-algebras with norm $\|\cdotp\|_{\mathbb{A}}$ and $\|\cdotp\|_{\mathbb{B}}$, respectively. We recall a fundamental result in fixed point theory. The following is the definition of a generalized metric space which was introduced by Luxemburg in 1958 [11].

### Definition 1.3

([11])

Let *X* be a set. A function $d: X\times X \to[0,\infty]$ is called a generalized metric on *X* if *d* satisfies the following conditions:

(i) $d(x,y) = 0$ if and only if $x=y$,
(i) $d(x,y) = d(y,x)$, for all $x,y \in X$,
(iii) $d(x,z) \leq d(x,y) + d(y,z)$, for all $x,y,z \in X$.

The following fixed point theorem will play important roles in proving our main results.

### Theorem 1.4

([9])

*Let* $(X,d)$ *be a complete generalized metric space and* $T : X\to X$ *be a strictly contractive mapping*, *that is*,

$$ d(Tx,Ty) \leq k d(x,y) $$

*for all* $x,y \in X$ *and for some Lipschitz* $k < 1$. *Then*, *for each given element* $x \in X$, *either*

$$ d\bigl(T^{n}x,T^{n+1}x\bigr) = \infty $$

*for all nonnegative integer* *n* *or there exists a positive integer* $n_{0}$ *such that*

(i) $d(T^{n}x,T^{n+1}x) < \infty$ *for all* $n \geq n_{0}$,
(ii) *the sequence* $\{ T^{n} x\}$ *converges to a fixed point* $y^{*}$ *of* *T*,
(iii) $y^{*}$ *is the unique fixed point of* *T* *in the set* $Y = \{ y \in X \mid d(T^{n_{0}}x,y) < \infty\}$,
(iv) $d(y,y^{*}) \leq \frac{1}{1-k} d(y,Ty)$, *for all* $y \in Y$.

The following lemma is useful for proving our main results.

### Lemma 1.5

([12])

*Let* $f : \mathbb{A} \to\mathbb{B}$ *be an additive mapping such that* $f(\mu x)=\mu f(x)$ *for all* $x\in\mathbb{A}$ *and all* $\mu\in\mathbb {S} := \{ \lambda\in\mathbb{C} \mid |\lambda| =1\}$. *Then the mapping* *f* *is* $\mathbb{C}$-*linear*.

## Stability of $C^{*}$-algebra homomorphisms

Let *f* be a mapping of $\mathbb{A}$ into $\mathbb{B}$. We define

2.1 $$ E_{\mu}f(x,y,z) := \alpha\mu f \biggl(\frac{x+y}{\alpha}+z \biggr) - f(\mu x) - f(\mu y) -\alpha f(\mu z), $$

for all $\mu\in\mathbb{S}$, for all $x,y,z \in\mathbb{A}$ and for any fixed positive integer $\alpha\geq2$.

We prove the Hyers–Ulam–Rassias stability of $C^{*}$-algebra homomorphisms for the functional equation $E_{\mu}f(x,y,z) = 0$.

### Theorem 2.1

*Let* $\phi: \mathbb{A}^{3} \to[0,\infty )$ *be a function such that there exists a* $k<1$ *satisfying*

2.2 $$ \phi(x,y,z) \leq\frac{2+\alpha}{\alpha} k \phi \biggl( \frac{\alpha }{2+\alpha}x, \frac{\alpha}{2+\alpha}y, \frac{\alpha}{2+\alpha}z \biggr) $$

*for all* $x,y,z \in\mathbb{A}$. *Let* *f* *be a mapping of* $\mathbb{A}$ *into* $\mathbb{B}$ *satisfying*

2.3 $$\begin{aligned}& \bigl\Vert E_{\mu}f(x,y,z) \bigr\Vert _{\mathbb{B}} \leq\phi(x,y,z), \end{aligned}$$

2.4 $$\begin{aligned}& \bigl\Vert f(xy) - f(x)f(y) \bigr\Vert _{\mathbb{B}} \leq \phi(x,y,0), \end{aligned}$$

2.5 $$\begin{aligned}& \bigl\Vert f\bigl(x^{*}\bigr) - f(x)^{*} \bigr\Vert _{\mathbb{B}} \leq\phi(x,x,x) , \end{aligned}$$

*for all* $\mu\in\mathbb{S}$ *and for all* $x,y,z \in\mathbb{A}$. *Then there exists a unique* $C^{*}$-*algebra homomorphism* $F : \mathbb{A} \to \mathbb{B}$ *such that*

2.6 $$ \bigl\Vert f(x) - F(x) \bigr\Vert _{\mathbb{B}} \leq \frac{1}{(1-k)(2+\alpha)} \phi(x,x,x) $$

*for all* $x \in\mathbb{A}$.

### Proof

Consider the set

$$ X:=\{ g \mid \mathbb{A} \to\mathbb{B}\} $$

and introduce the generalized metric on *X* as follows:

2.7 $$ d(g,h) = \inf\bigl\{ M \in (0,\infty) \mid \bigl\Vert g(x) - h(x) \bigr\Vert _{\mathbb{B}} \leq M\phi(x,x,x), \forall x \in\mathbb{A} \bigr\} . $$

It is easy to show that $(X,d)$ is complete.

Now, we consider the linear mapping $T : X \to X$ such that

$$ Tg(x) := \frac{\alpha}{2+\alpha}g \biggl( \frac{2+\alpha}{\alpha} x \biggr) $$

for all $x \in\mathbb{A}$. Next, we will show that *T* is a strictly contractive self-mapping of *X* with the Lipschitz constant *k*. For any $g,h \in X$, let $d(g,h) = K$ for some $K \in\mathbb{R}_{+}$. Then we have

$$\begin{aligned} & \bigl\Vert g(x) -h(x) \bigr\Vert _{\mathbb{B}} \leq K \phi(x,x,x) \quad\forall x \in \mathbb{A}, \\ &\quad \Rightarrow\quad \biggl\Vert g \biggl( \frac{2+\alpha}{\alpha}x \biggr) -h \biggl( \frac{2+\alpha}{\alpha}x \biggr) \biggr\Vert _{\mathbb{B}} \leq K \phi \biggl( \frac{2+\alpha}{\alpha}x, \frac{2+\alpha}{\alpha}x, \frac{2+\alpha}{\alpha}x \biggr) \quad\forall x \in\mathbb{A}, \\ &\quad \Rightarrow\quad \biggl\Vert \frac{\alpha}{2+\alpha}g \biggl( \frac{2+\alpha }{\alpha}x \biggr) - \frac{\alpha}{2+\alpha}h \biggl( \frac{2+\alpha }{\alpha}x \biggr) \biggr\Vert _{\mathbb{B}} \\ &\hphantom{\quad \Rightarrow\quad }\quad \leq \frac{\alpha}{2+\alpha}K \phi \biggl( \frac{2+\alpha}{\alpha}x, \frac{2+\alpha}{\alpha}x, \frac{2+\alpha}{\alpha}x \biggr) \quad\forall x \in\mathbb{A}. \end{aligned}$$

By (2.2), we obtain

$$\begin{aligned} & \bigl\Vert Tg(x) -Th(x) \bigr\Vert _{\mathbb{B}} \\ &\quad \leq\frac{\alpha}{2+\alpha}K \frac{2+\alpha}{\alpha} k \phi \biggl( \frac{\alpha}{2+\alpha}\cdotp \frac{2+\alpha}{\alpha} x, \frac{\alpha }{2+\alpha}\cdotp\frac{2+\alpha}{\alpha} x, \frac{\alpha}{2+\alpha }\cdotp\frac{2+\alpha}{\alpha} x \biggr) \\ &\quad \Rightarrow\quad \bigl\Vert Tg(x) -Th(x) \bigr\Vert _{\mathbb{B}} \leq Kk \phi (x,x,x) \quad\forall x \in\mathbb{A}. \\ &\quad \Rightarrow\quad d(Tg,Th) \leq Kk. \end{aligned}$$

Hence, we obtain

$$d(Tg,Th) \leq k d(g,h). $$

Letting $\mu= 1$ and $x=y=z$ in (2.1), we get

$$\begin{aligned} E_{\mu}f(x, x, x) & = \alpha f \biggl( \frac{x + x}{\alpha} + x \biggr) - f(x) -f(x) - \alpha f(x) =\alpha f \biggl( \frac{2+\alpha}{\alpha} x \biggr) - (2+ \alpha)f(x) \end{aligned}$$

for all $x \in\mathbb{A}$. By (2.3), we have

$$\bigl\Vert E_{\mu}f( x, x, x) \bigr\Vert _{\mathbb{B}} = \biggl\Vert \alpha f \biggl( \frac{2+\alpha}{\alpha} x \biggr) - (2+\alpha )f(x) \biggr\Vert _{\mathbb{B}} \leq \phi(x , x, x), $$

which implies that

$$\begin{aligned} \biggl\Vert f(x) - \frac{\alpha}{2+\alpha} f \biggl( \frac{2+\alpha}{\alpha }x \biggr) \biggr\Vert _{\mathbb{B}} &\leq\frac{1}{2+\alpha} \phi(x , x, x) \end{aligned}$$

for all $x \in\mathbb{A}$, that is,

$$\bigl\Vert f(x) - Tf(x) \bigr\Vert _{\mathbb{B}} \leq\frac{1}{2+\alpha} \phi(x,x,x) $$

for all $x \in\mathbb{A}$. It follows from (2.7) that we have

$$ d(f,Tf) \leq\frac{1}{2+\alpha}. $$

By Theorem 1.4, there exists a mapping $F : \mathbb{A} \to \mathbb{B}$ such that the following conditions hold.

1. *F* is a fixed point of *T*, that is, $TF(x)= F(x)$ for all $x \in\mathbb{A}$. Then we have
  $$F(x) = TF(x)= \frac{\alpha}{2+\alpha} F \biggl(\frac{2+\alpha}{\alpha }x \biggr)\quad \Rightarrow\quad F \biggl(\frac{2+\alpha}{\alpha}x \biggr) = \frac {2+\alpha}{\alpha}F(x) $$
  for all $x \in\mathbb{A}$. Moreover, the mapping *F* is a unique fixed point of *T* in the set
  $$ Y = \bigl\{ g \in X \mid d(f,g) < \infty\bigr\} . $$
  From (2.7), there exists $C \in(0,\infty)$ satisfying
  $$ \bigl\Vert f(x)-F(x) \bigr\Vert _{\mathbb{B}} \leq C \phi(x,x,x), $$
  for all $x \in\mathbb{A}$.
2. The sequence $\{T^{n}f\}$ converges to *F*. This implies that we have the equality
  2.8 $$ F(x)=\lim_{n\to\infty} \biggl( \frac{\alpha}{2+\alpha} \biggr)^{n} f \biggl( \biggl(\frac{2+\alpha}{\alpha} \biggr)^{n} x \biggr) $$
  for all $x \in\mathbb{A}$.
3. We obtain $d(f,F) \leq \frac{1}{1-k} d(f,Tf)$, which implies that
  2.9 $$ d(f,F) \leq \frac{1}{1-k} d(f,Tf) \leq\frac{1}{(1-k)(2+\alpha)}. $$
  Therefore, inequality (2.6) holds.

From (2.2), for any $j \in\mathbb{N}$, we have

$$\begin{aligned} & \biggl(\frac{\alpha}{2+\alpha} \biggr)^{j} \cdotp\phi \biggl( \biggl( \frac {2+\alpha}{\alpha} \biggr)^{j}x, \biggl(\frac{2+\alpha}{\alpha} \biggr)^{j}y, \biggl(\frac{2+\alpha}{\alpha} \biggr)^{j}z \biggr) \\ &\quad \leq \biggl(\frac{\alpha}{2+\alpha} \biggr)^{j} \cdotp \biggl( \frac {2+\alpha}{\alpha} \biggr) k \phi \biggl( \frac{\alpha}{2+\alpha} \biggl( \frac{2+\alpha}{\alpha} \biggr)^{j}x, \frac{\alpha}{2+\alpha} \biggl( \frac{2+\alpha}{\alpha} \biggr)^{j}y, \frac{\alpha}{2+\alpha} \biggl( \frac{2+\alpha}{\alpha} \biggr)^{j}z \biggr) \\ &\quad = k \biggl(\frac{\alpha}{2+\alpha} \biggr)^{j-1} \phi \biggl( \biggl( \frac {2+\alpha}{\alpha} \biggr)^{j-1}x, \biggl(\frac{2+\alpha}{\alpha} \biggr)^{j-1}y, \biggl(\frac{2+\alpha}{\alpha} \biggr)^{j-1}z \biggr) \\ &\quad \leq k \biggl(\frac{\alpha}{2+\alpha} \biggr)^{j-1} \biggl( \frac{2+\alpha }{\alpha} \biggr) k \phi \biggl( \frac{\alpha}{2+\alpha} \biggl( \frac {2+\alpha}{\alpha} \biggr)^{j-1}x,\\ &\qquad {} \frac{\alpha}{2+\alpha} \biggl( \frac {2+\alpha}{\alpha} \biggr)^{j-1}y, \frac{\alpha}{2+\alpha} \biggl( \frac {2+\alpha}{\alpha} \biggr)^{j-1}z \biggr) \\ &\quad = k^{2} \biggl(\frac{\alpha}{2+\alpha} \biggr)^{j-2} \phi \biggl( \biggl(\frac{2+\alpha}{\alpha} \biggr)^{j-2}x, \biggl(\frac{2+\alpha}{\alpha } \biggr)^{j-2}y, \biggl(\frac{2+\alpha}{\alpha} \biggr)^{j-2}z \biggr) \\ &\quad \leq\cdots\leq k^{j} \phi ( x, y,z ) \end{aligned}$$

for all $x,y,z \in\mathbb{A}$. Since $0 < k < 1$, we obtain

2.10 $$ \lim_{j\to\infty} \biggl(\frac{\alpha}{2+\alpha} \biggr)^{j} \cdotp\phi \biggl( \biggl(\frac{2+\alpha}{\alpha} \biggr)^{j}x, \biggl(\frac{2+\alpha }{\alpha} \biggr)^{j}y, \biggl(\frac{2+\alpha}{\alpha} \biggr)^{j}z \biggr) = 0 $$

for all $x,y,z \in\mathbb{A}$.

It follows from (2.3), (2.8) and (2.10) that

$$\begin{aligned} & \biggl\Vert \alpha F \biggl(\frac{x+y}{\alpha} + z \biggr) -F(x) - F(y) - \alpha F(z) \biggr\Vert _{\mathbb{B}} \\ &\quad = \biggl\Vert \alpha\lim_{n\to\infty} \biggl( \frac{\alpha}{2+\alpha } \biggr)^{n} f \biggl( \biggl(\frac{2+\alpha}{\alpha} \biggr)^{n} \biggl(\frac {x+y}{\alpha} + z \biggr) \biggr) -\lim_{n\to\infty} \biggl( \frac{\alpha}{2+\alpha} \biggr)^{n} f \biggl( \biggl(\frac{2+\alpha}{\alpha} \biggr)^{n} x \biggr) \\ &\qquad{}- \lim_{n\to\infty} \biggl( \frac{\alpha}{2+\alpha} \biggr)^{n} f \biggl( \biggl(\frac{2+\alpha}{\alpha} \biggr)^{n} y \biggr)-\alpha\lim_{n\to\infty} \biggl( \frac{\alpha}{2+\alpha} \biggr)^{n} f \biggl( \biggl(\frac{2+\alpha}{\alpha} \biggr)^{n} z \biggr) \biggr\Vert _{\mathbb{B}} \\ &\quad = \lim_{n\to\infty} \biggl( \frac{\alpha}{2+\alpha} \biggr)^{n} \biggl\Vert \alpha f \biggl(\frac{ (\frac{2+\alpha}{\alpha} )^{n}x+ (\frac {2+\alpha}{\alpha} )^{n}y}{\alpha} + \biggl(\frac{2+\alpha}{\alpha } \biggr)^{n}z \biggr) - f \biggl( \biggl(\frac{2+\alpha}{\alpha} \biggr)^{n} x \biggr) \\ &\qquad{}- f \biggl( \biggl(\frac{2+\alpha}{\alpha} \biggr)^{n} y \biggr)- \alpha f \biggl( \biggl(\frac{2+\alpha}{\alpha} \biggr)^{n} z \biggr) \biggr\Vert _{\mathbb{B}} \\ &\quad \leq \lim_{n\to\infty} \biggl( \frac{\alpha}{2+\alpha} \biggr)^{n}\phi \biggl( \biggl( \frac{2+\alpha}{\alpha} \biggr)^{n}x, \biggl( \frac{2+\alpha }{\alpha} \biggr)^{n}y, \biggl( \frac{2+\alpha}{\alpha} \biggr)^{n}z \biggr)=0 \end{aligned}$$

for all $x,y,z \in\mathbb{A}$. Hence, we have

2.11 $$ \alpha F \biggl(\frac{x+y}{\alpha} + z \biggr) = F(x) + F(y) + \alpha F(z) $$

for all $x,y,z \in\mathbb{A}$. From Corollary 1.2 and (2.11), we see that *F* is additive, that is,

2.12 $$ F(x + y) = F(x) + F(y) $$

for all $x,y \in\mathbb{A}$. Next, we can show that $F : \mathbb{A} \to\mathbb{B}$ is $\mathbb{C}$-linear. Firstly, we will show that, for any $x \in\mathbb{A}$, $F(\mu x) = \mu F(x)$ for all $\mu\in\mathbb {S}$. For each $\mu\in\mathbb{S}$, substituting $x,y,z$ in (2.1) by $( \frac{2+\alpha}{\alpha } )^{n} x$, we obtain

$$\begin{aligned} & E_{\mu}f \biggl( \biggl( \frac{2+\alpha}{\alpha} \biggr)^{n} x, \biggl( \frac {2+\alpha}{\alpha} \biggr)^{n} x, \biggl( \frac{2+\alpha}{\alpha} \biggr)^{n} x \biggr) \\ &\quad = \alpha\mu f \biggl(\frac{ ( \frac{2+\alpha}{\alpha} )^{n} x+ ( \frac{2+\alpha}{\alpha} )^{n} x}{\alpha}+ \biggl( \frac {2+\alpha}{\alpha} \biggr)^{n} x \biggr) - f \biggl(\mu \biggl( \frac{2+\alpha}{\alpha} \biggr)^{n} x \biggr) - f \biggl(\mu \biggl( \frac{2+\alpha}{\alpha} \biggr)^{n} x \biggr) \\ &\qquad {} -\alpha f \biggl(\mu \biggl( \frac{2+\alpha}{\alpha} \biggr)^{n} x \biggr) \\ &\quad = \alpha\mu f \biggl(\frac{(2+\alpha)}{\alpha}\cdotp \biggl( \frac {2+\alpha}{\alpha} \biggr)^{n} x \biggr) - (2+\alpha) f \biggl(\mu \biggl( \frac{2+\alpha}{\alpha} \biggr)^{n} x \biggr) \end{aligned}$$

for all $x \in\mathbb{A}$. By (2.3), we have

2.13 $$\begin{aligned} & \biggl\Vert E_{\mu}f \biggl( \biggl( \frac{2+\alpha}{\alpha} \biggr)^{n} x, \biggl( \frac{2+\alpha}{\alpha} \biggr)^{n} x, \biggl( \frac{2+\alpha}{\alpha} \biggr)^{n} x \biggr) \biggr\Vert _{\mathbb{B}} \\ &\quad = \biggl\Vert \alpha\mu f \biggl(\frac{2+\alpha}{\alpha}\cdotp \biggl( \frac {2+\alpha}{\alpha} \biggr)^{n} x \biggr) - (2+\alpha) f \biggl(\mu \biggl( \frac{2+\alpha}{\alpha} \biggr)^{n} x \biggr) \biggr\Vert _{\mathbb{B}} \\ &\quad \leq\phi \biggl( \biggl( \frac{2+\alpha}{\alpha} \biggr)^{n} x, \biggl( \frac {2+\alpha}{\alpha} \biggr)^{n} x, \biggl( \frac{2+\alpha}{\alpha} \biggr)^{n} x \biggr) \end{aligned}$$

for all $x \in\mathbb{A}$. From (2.13), in the case $\mu=1$, we obtain the fact that

2.14 $$\begin{aligned} & \biggl\Vert \alpha f \biggl(\frac{(2+\alpha)}{\alpha}\cdotp \biggl( \frac {2+\alpha}{\alpha} \biggr)^{n} x \biggr) - (2+\alpha) f \biggl( \biggl( \frac {2+\alpha}{\alpha} \biggr)^{n} x \biggr) \biggr\Vert _{\mathbb{B}} \\ &\quad \leq\phi \biggl( \biggl( \frac{2+\alpha}{\alpha} \biggr)^{n} x, \biggl( \frac {2+\alpha}{\alpha} \biggr)^{n} x, \biggl( \frac{2+\alpha}{\alpha} \biggr)^{n} x \biggr) \end{aligned}$$

for all $x \in\mathbb{A}$. It follows from (2.3), (2.13) and (2.14) that

$$\begin{aligned} & \biggl\Vert (2+\alpha) f \biggl( \mu \biggl( \frac{2+\alpha}{\alpha} \biggr)^{n} x \biggr) - (2+\alpha) \mu f \biggl( \biggl( \frac{2+\alpha}{\alpha } \biggr)^{n} x \biggr) \biggr\Vert _{\mathbb{B}} \\ &\quad = \biggl\Vert (2+\alpha) f \biggl( \mu \biggl( \frac{2+\alpha}{\alpha} \biggr)^{n} x \biggr) - \alpha\mu f \biggl(\frac{2+\alpha}{\alpha}\cdotp \biggl( \frac{2+\alpha }{\alpha} \biggr)^{n} x \biggr) \\ &\qquad{}+ \alpha\mu f \biggl(\frac{2+\alpha}{\alpha}\cdotp \biggl( \frac {2+\alpha}{\alpha} \biggr)^{n} x \biggr) - (2+\alpha) \mu f \biggl( \biggl( \frac{2+\alpha}{\alpha} \biggr)^{n} x \biggr) \biggr\Vert _{\mathbb{B}} \\ &\quad \leq \biggl\Vert (2+\alpha) f \biggl( \mu \biggl( \frac{2+\alpha}{\alpha } \biggr)^{n} x \biggr) - \alpha\mu f \biggl(\frac{2+\alpha}{\alpha}\cdotp \biggl( \frac{2+\alpha }{\alpha} \biggr)^{n} x \biggr) \biggr\Vert _{\mathbb{B}} \\ &\qquad{}+ \biggl\Vert \alpha\mu f \biggl(\frac{2+\alpha}{\alpha}\cdotp \biggl( \frac {2+\alpha}{\alpha} \biggr)^{n} x \biggr) - (2+\alpha) \mu f \biggl( \biggl( \frac{2+\alpha}{\alpha} \biggr)^{n} x \biggr) \biggr\Vert _{\mathbb{B}} \\ & \quad \leq \biggl\Vert (2+\alpha) f \biggl( \mu \biggl( \frac{2+\alpha}{\alpha } \biggr)^{n} x \biggr) - \alpha\mu f \biggl(\frac{2+\alpha}{\alpha}\cdotp \biggl( \frac{2+\alpha }{\alpha} \biggr)^{n} x \biggr) \biggr\Vert _{\mathbb{B}} \\ &\qquad{}+ \vert \mu \vert \biggl\Vert \alpha f \biggl(\frac{2+\alpha}{\alpha}\cdotp \biggl( \frac {2+\alpha}{\alpha} \biggr)^{n} x \biggr) - (2+\alpha) f \biggl( \biggl( \frac{2+\alpha}{\alpha} \biggr)^{n} x \biggr) \biggr\Vert _{\mathbb{B}} \\ & \quad \leq2 \phi \biggl( \biggl( \frac{2+\alpha}{\alpha} \biggr)^{n} x, \biggl( \frac{2+\alpha}{\alpha} \biggr)^{n} x, \biggl( \frac{2+\alpha}{\alpha} \biggr)^{n} x \biggr) \end{aligned}$$

for all $x \in\mathbb{A}$. This implies that

$$\begin{aligned} & \biggl\Vert \biggl( \frac{\alpha}{2+\alpha} \biggr)^{n} f \biggl( \mu \biggl( \frac{2+\alpha}{\alpha} \biggr)^{n} x \biggr) - \biggl( \frac{\alpha }{2+\alpha} \biggr)^{n} \mu f \biggl( \biggl( \frac{2+\alpha}{\alpha} \biggr)^{n} x \biggr) \biggr\Vert _{\mathbb{B}} \\ & \quad \leq\frac{2}{2+\alpha} \biggl( \frac{\alpha}{2+\alpha} \biggr)^{n} \phi \biggl( \biggl( \frac{2+\alpha}{\alpha} \biggr)^{n} x, \biggl( \frac{2+\alpha }{\alpha} \biggr)^{n} x, \biggl( \frac{2+\alpha}{\alpha} \biggr)^{n} x \biggr) \\ &\quad \leq \biggl( \frac{\alpha}{2+\alpha} \biggr)^{n} \phi \biggl( \biggl( \frac {2+\alpha}{\alpha} \biggr)^{n} x, \biggl( \frac{2+\alpha}{\alpha} \biggr)^{n} x, \biggl( \frac{2+\alpha}{\alpha} \biggr)^{n} x \biggr) \end{aligned}$$

for all $x\in\mathbb{A}$. By (2.10), we have

$$\lim_{n \to\infty} \biggl\Vert \biggl( \frac{\alpha}{2+\alpha} \biggr)^{n} f \biggl( \mu \biggl( \frac{2+\alpha}{\alpha} \biggr)^{n} x \biggr) - \biggl( \frac{\alpha}{2+\alpha} \biggr)^{n} \mu f \biggl( \biggl( \frac{2+\alpha }{\alpha} \biggr)^{n} x \biggr) \biggr\Vert _{\mathbb{B}} = 0, $$

which implies that

2.15 $$\begin{aligned} F(\mu x) = \mu F(x) \end{aligned}$$

for all $x \in\mathbb{A}$. It follows from (2.12), (2.15) and Lemma 1.5 that $F:\mathbb{A} \to\mathbb {B}$ is $\mathbb{C}$-linear. Next, we will show that *F* is a $C^{*}$-algebra homomorphism. It follows from (2.4) that

$$\begin{aligned} & \bigl\Vert F(xy) -F(x)F(y) \bigr\Vert _{\mathbb{B}} \\ &\quad = \biggl\Vert \lim_{n\to\infty} \biggl( \frac{\alpha}{2+\alpha} \biggr)^{2n} f \biggl( \biggl(\frac{2+\alpha}{\alpha} \biggr)^{2n} xy \biggr) \\ &\qquad{}- \lim_{n\to\infty} \biggl( \frac{\alpha}{2+\alpha} \biggr)^{n} f \biggl( \biggl(\frac{2+\alpha}{\alpha} \biggr)^{n} x \biggr) \cdotp\lim_{n\to\infty} \biggl( \frac{\alpha}{2+\alpha} \biggr)^{n} f \biggl( \biggl(\frac{2+\alpha}{\alpha} \biggr)^{n}y \biggr) \biggr\Vert _{\mathbb{B}} \\ &\quad = \lim_{n\to\infty} \biggl( \frac{\alpha}{2+\alpha} \biggr)^{2n} \biggl\Vert f \biggl( \biggl(\frac{2+\alpha}{\alpha} \biggr)^{2n} xy \biggr) - f \biggl( \biggl(\frac{2+\alpha}{\alpha} \biggr)^{n} x \biggr) f \biggl( \biggl(\frac {2+\alpha}{\alpha} \biggr)^{n} y \biggr) \biggr\Vert _{\mathbb{B}} \\ & \quad \leq\lim_{n\to\infty} \biggl( \frac{\alpha}{2+\alpha} \biggr)^{2n} \phi \biggl( \biggl(\frac{2+\alpha}{\alpha} \biggr)^{n}x, \biggl(\frac {2+\alpha}{\alpha} \biggr)^{n} y, 0 \biggr) \\ &\quad \leq\lim_{n\to\infty} \biggl( \frac{\alpha}{2+\alpha} \biggr)^{n} \phi \biggl( \biggl(\frac{2+\alpha}{\alpha} \biggr)^{n}x, \biggl(\frac {2+\alpha}{\alpha} \biggr)^{n} y, 0 \biggr) = 0 \end{aligned}$$

for all $x,y \in\mathbb{A}$. Hence

$$F(xy) = F(x)F(y) $$

for all $x,y \in\mathbb{A}$.

Finally, it follows from (2.5) that

$$\begin{aligned} & \bigl\Vert F\bigl(x^{*}\bigr) - \bigl(F(x)\bigr)^{*} \bigr\Vert _{\mathbb{B}} \\ &\quad = \biggl\Vert \lim_{n\to\infty} \biggl( \frac{\alpha}{2+\alpha} \biggr)^{n} f \biggl( \biggl(\frac{2+\alpha}{\alpha} \biggr)^{n} x^{*} \biggr) - \biggl( \lim_{n\to\infty} \biggl( \frac{\alpha}{2+\alpha} \biggr)^{n} f \biggl( \biggl(\frac{2+\alpha}{\alpha} \biggr)^{n} x \biggr) \biggr)^{*} \biggr\Vert _{\mathbb{B}} \\ &\quad = \biggl\Vert \lim_{n\to\infty} \biggl( \frac{\alpha}{2+\alpha} \biggr)^{n} f \biggl( \biggl(\frac{2+\alpha}{\alpha} \biggr)^{n} x^{*} \biggr) - \lim_{n\to\infty} \biggl( \biggl( \frac{\alpha}{2+\alpha} \biggr)^{n} f \biggl( \biggl(\frac{2+\alpha}{\alpha} \biggr)^{n} x \biggr) \biggr)^{*} \biggr\Vert _{\mathbb{B}} \\ &\quad = \biggl\Vert \lim_{n\to\infty} \biggl( \frac{\alpha}{2+\alpha} \biggr)^{n} f \biggl( \biggl( \biggl(\frac{2+\alpha}{\alpha} \biggr)^{n} x \biggr)^{*} \biggr) - \lim_{n\to\infty} \biggl( \frac{\alpha}{2+\alpha} \biggr)^{n} \biggl(f \biggl( \biggl(\frac{2+\alpha}{\alpha} \biggr)^{n} x \biggr) \biggr)^{*} \biggr\Vert _{\mathbb{B}} \\ &\quad = \lim_{n\to\infty} \biggl( \frac{\alpha}{2+\alpha} \biggr)^{n} \biggl\Vert f \biggl( \biggl( \biggl(\frac{2+\alpha}{\alpha} \biggr)^{n} x \biggr)^{*} \biggr) - \biggl(f \biggl( \biggl(\frac{2+\alpha}{\alpha} \biggr)^{n} x \biggr) \biggr)^{*} \biggr\Vert _{\mathbb{B}} \\ &\quad \leq\lim_{n \to\infty} \biggl( \frac{\alpha}{2+\alpha} \biggr)^{n} \phi \biggl( \biggl(\frac{2+\alpha}{\alpha} \biggr)^{n} x, \biggl(\frac{2+\alpha }{\alpha} \biggr)^{n} x, \biggl(\frac{2+\alpha}{\alpha} \biggr)^{n} x \biggr) = 0 \end{aligned}$$

for all $x\in\mathbb{A}$, which implies that

$$F\bigl(x^{*}\bigr) = \bigl( F(x) \bigr)^{*} $$

for all $x \in\mathbb{A}$. Therefore, $F : \mathbb{A} \to\mathbb{B}$ is a $C^{*}$-algebra homomorphism. □

### Corollary 2.2

*Let* $p\in[0,1)$, $\varepsilon\in[0,\infty)$ *and* *f* *be a mapping of* $\mathbb{A}$ *into* $\mathbb{B}$ *such that*

2.16 $$\begin{aligned}& \bigl\Vert E_{\mu} f(x,y,z) \bigr\Vert _{\mathbb{B}} \leq\varepsilon \bigl( \Vert x \Vert _{\mathbb{A}}^{p} + \Vert y \Vert _{\mathbb{A}}^{p} + \Vert z \Vert _{\mathbb{A}}^{p} \bigr), \end{aligned}$$

2.17 $$\begin{aligned}& \bigl\Vert f(xy) - f(x)f(y) \bigr\Vert _{\mathbb{B}} \leq \varepsilon\bigl( \Vert x \Vert _{\mathbb {A}}^{p} + \Vert y \Vert _{\mathbb{A}}^{p}\bigr), \end{aligned}$$

2.18 $$\begin{aligned}& \bigl\Vert f\bigl(x^{*}\bigr) - f(x)^{*} \bigr\Vert _{\mathbb{B}} \leq3\varepsilon \Vert x \Vert _{\mathbb{A}}^{p} \end{aligned}$$

*for all* $\mu\in\mathbb{S}$ *and for all* $x,y,z \in\mathbb{A}$. *Then there exists a unique* $C^{*}$-*algebra homomorphism* $F : \mathbb{A} \to \mathbb{B}$ *such that*

$$ \bigl\Vert f(x) - F(x) \bigr\Vert _{\mathbb{B}} \leq\frac{3\varepsilon}{ (1- (\frac{2+\alpha}{\alpha} )^{p-1} )(2+\alpha)} \Vert x \Vert _{\mathbb{A}}^{p} $$

*for all* $x \in\mathbb{A}$.

### Proof

The proof follows from Theorem 2.1 by taking

$$\phi (x,y,z ) = \theta\bigl( \Vert x \Vert _{\mathbb{A}}^{p}+ \Vert y \Vert _{\mathbb {A}}^{p}+ \Vert z \Vert _{\mathbb{A}}^{p}\bigr) $$

for all $x,y,z \in\mathbb{A}$. Then $k= (\frac{2+\alpha}{\alpha } )^{p-1}$ and we get the desired results. □

### Theorem 2.3

*Let* $\phi: \mathbb{A}^{3} \to[0,\infty)$ *be a function such that there exists a* $k < 1$ *such that*

2.19 $$ \phi(x,y,z) \leq \biggl(\frac{\alpha}{2+\alpha} \biggr)^{2} k \phi \biggl( \frac{2+\alpha}{\alpha}x, \frac{2+\alpha}{\alpha}y, \frac{2+\alpha }{\alpha}z \biggr) $$

*for all* $x,y,z \in\mathbb{A}$. *Let* *f* *be a mapping of* $\mathbb{A}$ *into* $\mathbb{B}$ *satisfying* (2.3), (2.4) *and* (2.5). *Then there exists a unique* $C^{*}$-*algebra homomorphism* $F : \mathbb{A} \to\mathbb{B}$ *such that*

2.20 $$ \bigl\Vert f(x) - F(x) \bigr\Vert _{\mathbb{B}} \leq \frac{\alpha k}{(1-k)(2+\alpha)^{2}} \phi(x,x,x) $$

*for all* $x \in\mathbb{A}$.

### Proof

We consider the linear mapping $T : X\to X$ such that

2.21 $$ Tg(x) :=\frac{2+\alpha}{\alpha} g \biggl( \frac{\alpha}{2+\alpha}x \biggr) $$

for all $x \in\mathbb{A}$. By a similar proof to Theorem 2.1, *T* is a strictly contractive self-mapping of *X* with the Lipschitz constant *k*. Letting $\mu= 1$ and substituting $x,y,z$ in (2.3) by $\frac {\alpha}{2+\alpha}x$, we have

2.22 $$\begin{aligned} \biggl\Vert E_{\mu}f \biggl(\frac{\alpha}{2+\alpha}x, \frac{\alpha}{2+\alpha }x, \frac{\alpha}{2+\alpha}x \biggr) \biggr\Vert _{\mathbb{B}} &= \biggl\Vert \alpha f (x ) - (2+\alpha)f \biggl( \frac{\alpha }{2+\alpha}x \biggr) \biggr\Vert _{\mathbb{B}} \\ &\leq \phi \biggl(\frac{\alpha}{2+\alpha}x, \frac{\alpha}{2+\alpha}x, \frac{\alpha}{2+\alpha}x \biggr) \end{aligned}$$

for all $x\in\mathbb{A}$. From inequality (2.22) we get

$$\begin{aligned} &\biggl\Vert f (x ) - \frac{2+\alpha}{\alpha}f \biggl(\frac{\alpha }{2+\alpha}x \biggr) \biggr\Vert _{\mathbb{B}}\\ &\quad \leq \frac{1}{\alpha}\phi \biggl( \frac{\alpha}{2+\alpha}x, \frac {\alpha}{2+\alpha}x, \frac{\alpha}{2+\alpha}x \biggr) \\ &\quad \leq\frac{1}{\alpha} \cdotp \biggl( \frac{\alpha}{2+\alpha} \biggr)^{2} k \phi \biggl(\frac{2+\alpha}{\alpha}\cdotp\frac{\alpha}{2+\alpha}x, \frac{2+\alpha}{\alpha}\cdotp \frac{\alpha}{2+\alpha}x, \frac{2+\alpha }{\alpha}\cdotp\frac{\alpha}{2+\alpha}x \biggr) \\ &\quad = \frac{\alpha k}{(2+\alpha)^{2}} \cdotp\phi(x,x,x) \end{aligned}$$

for all $x \in\mathbb{A}$, that is,

$$\begin{aligned} \bigl\Vert Tf(x) - f(x) \bigr\Vert _{\mathbb{B}} &\leq \frac{\alpha k}{(2+\alpha)^{2}} \phi(x , x, x) \end{aligned}$$

for all $x \in\mathbb{A}$. Hence, we obtain

$$ d(f,Tf) \leq\frac{\alpha k}{(2+\alpha)^{2}}. $$

By Theorem 1.4, there exists a mapping $F : \mathbb{A} \to \mathbb{B}$ such that the following conditions hold.

1. *F* is a fixed point of *T*, that is, $TF(x) = F(x)$ for all $x \in\mathbb{A}$. Then we have
  $$F(x) = TF(x) = \frac{2+\alpha}{\alpha} F \biggl(\frac{\alpha}{2+\alpha }x \biggr) \quad \Rightarrow\quad F \biggl(\frac{\alpha}{2+\alpha}x \biggr) = \frac {\alpha}{2+\alpha}F(x) $$
  for all $x \in\mathbb{A}$. Moreover, the mapping *F* is a unique fixed point of *T* in the set
  $$ Y = \bigl\{ g \in X \mid d(f,g) < \infty\bigr\} . $$
  From (2.7), there exists $C \in(0,\infty)$ satisfying
  $$ \bigl\Vert f(x)-F(x) \bigr\Vert _{\mathbb{B}} \leq C \phi(x,x,x), $$
  for all $x \in\mathbb{A}$.
2. The sequence $\{T^{n}f\}$ converges to *F*. This implies that the equality
  2.23 $$ F(x)=\lim_{n\to\infty} \biggl( \frac{2+\alpha}{\alpha} \biggr)^{n} f \biggl( \biggl(\frac{\alpha}{2+\alpha} \biggr)^{n} x \biggr) $$
  for all $x \in\mathbb{A}$.
3. We obtain $d(f,F) \leq \frac{1}{1-k} d(f,Tf)$, which implies that
  $$ d(f,F) \leq \frac{1}{1-k} d(f,Tf) \leq\frac{\alpha k}{(1-k)(2+\alpha)^{2}}. $$
  Therefore, inequality (2.20) holds.

It follows from (2.19) and same argument in Theorem 2.1 that we obtain

2.24 $$ \lim_{j\to\infty} \biggl(\frac{2+\alpha}{\alpha} \biggr)^{2j} \cdotp\phi \biggl( \biggl(\frac{\alpha}{2+\alpha} \biggr)^{j}x, \biggl(\frac{\alpha }{2+\alpha} \biggr)^{j}y, \biggl( \frac{\alpha}{2+\alpha} \biggr)^{j}z \biggr) = 0 $$

for all $x,y,z \in\mathbb{A}$. It follows from (2.3), (2.23), (2.24) that

$$\begin{aligned} & \biggl\Vert \alpha F \biggl(\frac{x+y}{\alpha} + z \biggr) -F(x) - F(y) - \alpha F(z) \biggr\Vert _{\mathbb{B}} \\ &\quad = \biggl\Vert \alpha\lim_{n\to\infty} \biggl( \frac{2+\alpha}{\alpha } \biggr)^{n} f \biggl( \biggl(\frac{\alpha}{2+\alpha} \biggr)^{n} \biggl(\frac {x+y}{\alpha} + z \biggr) \biggr) -\lim_{n\to\infty} \biggl( \frac{2+\alpha}{\alpha} \biggr)^{n} f \biggl( \biggl(\frac{\alpha}{2+\alpha} \biggr)^{n} x \biggr) \\ &\qquad{}- \lim_{n\to\infty} \biggl( \frac{2+\alpha}{\alpha} \biggr)^{n} f \biggl( \biggl(\frac{\alpha}{2+\alpha} \biggr)^{n} y \biggr)-\alpha\lim_{n\to\infty} \biggl( \frac{2+\alpha}{\alpha} \biggr)^{n} f \biggl( \biggl(\frac{\alpha}{2+\alpha} \biggr)^{n} z \biggr) \biggr\Vert _{\mathbb{B}} \\ &\quad = \lim_{n\to\infty} \biggl( \frac{2+\alpha}{\alpha} \biggr)^{n} \biggl\Vert \alpha f \biggl(\frac{ (\frac{\alpha}{2+\alpha} )^{n}x+ (\frac {\alpha}{2+\alpha} )^{n}y}{\alpha} + \biggl(\frac{\alpha}{2+\alpha } \biggr)^{n}z \biggr) - f \biggl( \biggl(\frac{\alpha}{2+\alpha} \biggr)^{n} x \biggr) \\ &\qquad{}- f \biggl( \biggl(\frac{\alpha}{2+\alpha} \biggr)^{n} y \biggr)- \alpha f \biggl( \biggl(\frac{\alpha}{2+\alpha} \biggr)^{n} z \biggr) \biggr\Vert _{\mathbb{B}} \\ &\quad \leq \lim_{n\to\infty} \biggl( \frac{2+\alpha}{\alpha} \biggr)^{n}\phi \biggl( \biggl( \frac{\alpha}{2+\alpha} \biggr)^{n}x, \biggl( \frac{\alpha }{2+\alpha} \biggr)^{n}y, \biggl( \frac{\alpha}{2+\alpha} \biggr)^{n}z \biggr) \\ &\quad \leq \lim_{n\to\infty} \biggl( \frac{2+\alpha}{\alpha} \biggr)^{2n}\phi \biggl( \biggl( \frac{\alpha}{2+\alpha} \biggr)^{n}x, \biggl( \frac{\alpha }{2+\alpha} \biggr)^{n}y, \biggl( \frac{\alpha}{2+\alpha} \biggr)^{n}z \biggr)=0 \end{aligned}$$

for all $x,y,z \in\mathbb{A}$. Hence, we have

$$ \alpha F \biggl(\frac{x+y}{\alpha} + z \biggr) = F(x) + F(y) + \alpha F(z) $$

for all $x,y,z \in\mathbb{A}$. From Corollary 1.2 and the above equation, we see that *F* is additive for all $x,y \in\mathbb{A}$. Next, we can show that $F : \mathbb{A} \to\mathbb{B}$ is $\mathbb{C}$-linear. Firstly, we will show that, for any $x \in\mathbb{A}$, $F(\mu x) = \mu F(x)$ for all $\mu\in\mathbb {S}$. For each $\mu\in\mathbb{S}$, substituting $x,y,z$ in (2.1) by $( \frac{\alpha}{2+\alpha } )^{n} x$, we obtain

$$\begin{aligned} & E_{\mu}f \biggl( \biggl( \frac{\alpha}{2+\alpha} \biggr)^{n} x, \biggl( \frac {\alpha}{2+\alpha} \biggr)^{n} x, \biggl( \frac{\alpha}{2+\alpha} \biggr)^{n} x \biggr) \\ &\quad = \alpha\mu f \biggl(\frac{ ( \frac{\alpha}{2+\alpha} )^{n} x+ ( \frac{\alpha}{2+\alpha} )^{n} x}{\alpha}+ \biggl( \frac{\alpha }{2+\alpha} \biggr)^{n} x \biggr) - f \biggl(\mu \biggl( \frac{\alpha}{2+\alpha} \biggr)^{n} x \biggr) - f \biggl(\mu \biggl( \frac{\alpha}{2+\alpha} \biggr)^{n} x \biggr) \\ &\qquad {} -\alpha f \biggl(\mu \biggl( \frac{\alpha}{2+\alpha} \biggr)^{n} x \biggr) \\ &\quad = \alpha\mu f \biggl(\frac{2+\alpha}{\alpha} \biggl( \frac{\alpha }{2+\alpha} \biggr)^{n} x \biggr) - (2+\alpha) f \biggl(\mu \biggl( \frac {\alpha}{2+\alpha} \biggr)^{n} x \biggr) \end{aligned}$$

for all $x \in\mathbb{A}$. By (2.3), we have

2.25 $$\begin{aligned} & \biggl\Vert E_{\mu}f \biggl( \biggl( \frac{\alpha}{2+\alpha} \biggr)^{n} x, \biggl( \frac{\alpha}{2+\alpha} \biggr)^{n} x, \biggl( \frac{\alpha}{2+\alpha} \biggr)^{n} x \biggr) \biggr\Vert _{\mathbb{B}} \\ &\quad = \biggl\Vert \alpha\mu f \biggl(\frac{2+\alpha}{\alpha} \biggl( \frac{\alpha }{2+\alpha} \biggr)^{n} x \biggr) - (2+\alpha) f \biggl(\mu \biggl( \frac {\alpha}{2+\alpha} \biggr)^{n} x \biggr) \biggr\Vert _{\mathbb{B}} \\ &\quad \leq\phi \biggl( \biggl( \frac{\alpha}{2+\alpha} \biggr)^{n} x, \biggl( \frac {\alpha}{2+\alpha} \biggr)^{n} x, \biggl( \frac{\alpha}{2+\alpha} \biggr)^{n} x \biggr) \end{aligned}$$

for all $x \in\mathbb{A}$. From (2.25), in the case $\mu=1$, we obtain the fact that

2.26 $$\begin{aligned} & \biggl\Vert \alpha f \biggl(\frac{2+\alpha}{\alpha} \biggl( \frac{\alpha }{2+\alpha} \biggr)^{n} x \biggr) - (2+\alpha) f \biggl( \biggl( \frac{\alpha }{2+\alpha} \biggr)^{n} x \biggr) \biggr\Vert _{\mathbb{B}} \\ &\quad \leq\phi \biggl( \biggl( \frac{\alpha}{2+\alpha} \biggr)^{n} x, \biggl( \frac {\alpha}{2+\alpha} \biggr)^{n} x, \biggl( \frac{\alpha}{2+\alpha} \biggr)^{n} x \biggr) \end{aligned}$$

for all $x \in\mathbb{A}$. It follows from (2.3), (2.25) and (2.26) that

$$\begin{aligned} & \biggl\Vert (2+\alpha) f \biggl( \mu \biggl( \frac{\alpha}{2+\alpha} \biggr)^{n} x \biggr) - (2+\alpha) \mu f \biggl( \biggl( \frac{\alpha}{2+\alpha} \biggr)^{n} x \biggr) \biggr\Vert _{\mathbb{B}} \\ &\quad = \biggl\Vert (2+\alpha) f \biggl( \mu \biggl( \frac{\alpha}{2+\alpha} \biggr)^{n} x \biggr) - \alpha\mu f \biggl(\frac{2+\alpha}{\alpha} \biggl( \frac{\alpha }{2+\alpha} \biggr)^{n} x \biggr) \\ &\qquad{}+ \alpha\mu f \biggl(\frac{2+\alpha}{\alpha} \biggl( \frac{\alpha }{2+\alpha} \biggr)^{n} x \biggr) - (2+\alpha) \mu f \biggl( \biggl( \frac{\alpha}{2+\alpha} \biggr)^{n} x \biggr) \biggr\Vert _{\mathbb{B}} \\ & \quad \leq \biggl\Vert (2+\alpha) f \biggl( \mu \biggl( \frac{\alpha}{2+\alpha } \biggr)^{n} x \biggr) - \alpha\mu f \biggl(\frac{2+\alpha}{\alpha} \biggl( \frac{\alpha }{2+\alpha} \biggr)^{n} x \biggr) \biggr\Vert _{\mathbb{B}} \\ &\qquad{}+ \biggl\Vert \alpha\mu f \biggl(\frac{2+\alpha}{\alpha} \biggl( \frac{\alpha }{2+\alpha} \biggr)^{n} x \biggr) - (2+\alpha) \mu f \biggl( \biggl( \frac{\alpha}{2+\alpha} \biggr)^{n} x \biggr) \biggr\Vert _{\mathbb{B}} \\ &\quad = \biggl\Vert (2+\alpha) f \biggl( \mu \biggl( \frac{\alpha}{2+\alpha} \biggr)^{n} x \biggr) - \alpha\mu f \biggl(\frac{2+\alpha}{\alpha} \biggl( \frac{\alpha }{2+\alpha} \biggr)^{n} x \biggr) \biggr\Vert _{\mathbb{B}} \\ &\qquad{}+ \vert \mu \vert \biggl\Vert \alpha f \biggl(\frac{2+\alpha}{\alpha} \biggl( \frac{\alpha }{2+\alpha} \biggr)^{n} x \biggr) - (2+\alpha) f \biggl( \biggl( \frac{\alpha}{2+\alpha} \biggr)^{n} x \biggr) \biggr\Vert _{\mathbb{B}} \\ &\quad \leq2 \phi \biggl( \biggl( \frac{\alpha}{2+\alpha} \biggr)^{n} x, \biggl( \frac{\alpha}{2+\alpha} \biggr)^{n} x, \biggl( \frac{\alpha}{2+\alpha} \biggr)^{n} x \biggr) \end{aligned}$$

for all $x \in\mathbb{A}$. This implies that

$$\begin{aligned} & \biggl\Vert \biggl( \frac{2+\alpha}{\alpha} \biggr)^{n} f \biggl( \mu \biggl( \frac{\alpha}{2+\alpha} \biggr)^{n} x \biggr) - \biggl( \frac{2+\alpha }{\alpha} \biggr)^{n} \mu f \biggl( \biggl( \frac{\alpha}{2+\alpha} \biggr)^{n} x \biggr) \biggr\Vert _{\mathbb{B}} \\ & \quad \leq\frac{2}{2+\alpha} \biggl( \frac{2+\alpha}{\alpha} \biggr)^{n} \phi \biggl( \biggl( \frac{\alpha}{2+\alpha} \biggr)^{n} x, \biggl( \frac{\alpha }{2+\alpha} \biggr)^{n} x, \biggl( \frac{\alpha}{2+\alpha} \biggr)^{n} x \biggr) \\ &\quad \leq \biggl( \frac{2+\alpha}{\alpha} \biggr)^{n} \phi \biggl( \biggl( \frac {\alpha}{2+\alpha} \biggr)^{n} x, \biggl( \frac{\alpha}{2+\alpha} \biggr)^{n} x, \biggl( \frac{\alpha}{2+\alpha} \biggr)^{n} x \biggr) \\ &\quad \leq \biggl( \frac{2+\alpha}{\alpha} \biggr)^{2n} \phi \biggl( \biggl( \frac{\alpha}{2+\alpha} \biggr)^{n} x, \biggl( \frac{\alpha}{2+\alpha} \biggr)^{n} x, \biggl( \frac{\alpha}{2+\alpha} \biggr)^{n} x \biggr) \end{aligned}$$

for all $x\in\mathbb{A}$. By (2.24), we have

$$\lim_{n \to\infty} \biggl\Vert \biggl( \frac{2+\alpha}{\alpha} \biggr)^{n} f \biggl( \mu \biggl( \frac{\alpha}{2+\alpha} \biggr)^{n} x \biggr) - \biggl( \frac{2+\alpha}{\alpha} \biggr)^{n} \mu f \biggl( \biggl( \frac{\alpha }{2+\alpha} \biggr)^{n} x \biggr) \biggr\Vert _{\mathbb{B}} = 0, $$

which implies that

$$\begin{aligned} F(\mu x) = \mu F(x) \end{aligned}$$

for all $x \in\mathbb{A}$. By Lemma 1.5, we see that *F* is $\mathbb{C}$-linear. The fact that $F(xy) = F(x)F(y)$ and $F(x^{*})=F(x)^{*}$ for all $x,y \in\mathbb{A}$ can be obtained in a similar method as in the proof of Theorem 2.1. □

### Corollary 2.4

*Let* $p\in(2,\infty)$, $\varepsilon\in[0,\infty)$ *and* *f* *be a mapping of* $\mathbb{A}$ *into* $\mathbb{B}$ *satisfying* (2.16), (2.17) *and* (2.18). *Then there exists a unique* $C^{*}$-*algebra homomorphism* $F : \mathbb{A} \to\mathbb{B}$ *such that*

2.27 $$ \bigl\Vert f(x) - F(x) \bigr\Vert _{\mathbb{B}} \leq\frac{3\alpha\varepsilon }{ ( ( \frac{2+\alpha}{\alpha} )^{p-2} -1 )(2+\alpha )^{2}} \Vert x \Vert _{\mathbb{A}}^{p} $$

*for all* $x \in\mathbb{A}$.

### Proof

The proof follows from Theorem 2.3 and Corollary 2.2 by taking

$$\phi(x,y,z) = \varepsilon\bigl( \Vert x \Vert _{\mathbb{A}}^{p}+ \Vert y \Vert _{\mathbb{A}}^{p}+ \Vert z \Vert _{\mathbb{A}}^{p}\bigr) $$

for all $x,y,z \in\mathbb{A}$. Then $k= (\frac{\alpha}{2+\alpha } )^{p-2}$ and we get the desired results. □

### Remark 2.5

If $\alpha=2$, then Theorem 2.1, Corollary 2.2 and Theorem 2.3 we recover Theorem 2.1, Corollary 2.2 and Theorem 2.3 in [10], respectively.

## Stability of generalized *θ*-derivations on $C^{*}$-algebras

Let *f* be a mapping of $\mathbb{A}$ into $\mathbb{A}$. We define

$$ E_{\mu}f(x,y,z) := \alpha\mu f \biggl(\frac{x+y}{\alpha}+z \biggr) - f( \mu x) - f(\mu y) -\alpha f(\mu z), $$

for all $\mu\in\mathbb{S}$ and all $x,y,z \in\mathbb{A}$ and for any fixed positive integer $\alpha\geq2$.

### Definition 3.1

A generalized *θ*-derivation $\delta: \mathbb{A} \to\mathbb{A}$ is a $\mathbb{C}$-linear map satisfying

$$ \delta(xyz) = \delta(xy)\theta(z) - \theta(x)\delta(y)\theta(z)+ \theta (x) \delta(yz). $$

for all $x,y,z \in\mathbb{A}$, where $\theta: \mathbb{A} \to\mathbb {A}$ is a $\mathbb{C}$-linear mapping.

We prove the Hyers–Ulam–Rassias stability of generalized *θ*-derivation on $C^{*}$-algebras for the functional equation $E_{\mu} f(x,y,z) = 0$.

### Theorem 3.1

*Let* $\phi: \mathbb{A}^{3} \to[0,\infty)$ *be a function such that there exists a* $k < 1$ *satisfying* (2.2). *Let* $f, h$ *be mappings of* $\mathbb{A}$ *into itself satisfying*

3.1 $$\begin{aligned}& \bigl\Vert E_{\mu}f(x,y,z) \bigr\Vert _{\mathbb{A}} \leq\phi(x,y,z), \end{aligned}$$

3.2 $$\begin{aligned}& \bigl\Vert f(xyz) - f(xy)h(z) + h(x)f(y)h(z) - h(x)f(yz) \bigr\Vert _{\mathbb{A}} \leq \phi(x,y,z), \end{aligned}$$

3.3 $$\begin{aligned}& \biggl\Vert \mu h \biggl( \frac{2+\alpha}{2\alpha}(x+y) \biggr) - \frac {2+\alpha}{2\alpha} \bigl(h(\mu x) + h(\mu y) \bigr) \biggr\Vert _{\mathbb {A}} \leq\phi(x,y,x), \end{aligned}$$

3.4 $$\begin{aligned}& \bigl\Vert f\bigl(x^{*}\bigr) - f(x)^{*} \bigr\Vert _{\mathbb{A}} \leq\phi(x,x,x) , \end{aligned}$$

*for all* $\mu\in\mathbb{S}$ *and for all* $x,y,z \in\mathbb{A}$. *Then there exist unique* $\mathbb{C}$-*linear mappings* $\delta, \theta: \mathbb{A} \to\mathbb{A}$ *such that*

3.5 $$\begin{aligned}& \bigl\Vert f(x) - \delta(x) \bigr\Vert _{\mathbb{A}} \leq \frac{1}{(1-k)(2+\alpha)}\phi(x,x,x), \end{aligned}$$

3.6 $$\begin{aligned}& \bigl\Vert h(x) - \theta(x) \bigr\Vert _{\mathbb{A}} \leq \frac{\alpha}{(1-k)(2+\alpha )}\phi(x,x,x) , \end{aligned}$$

*for all* $x \in\mathbb{A}$. *Moreover*, $\delta: \mathbb{A} \to\mathbb {A}$ *is a generalized* *θ*-*derivation on* $\mathbb{A}$.

### Proof

Let $(X,d)$ be the generalized metric space as in the proof of Theorem 2.1. We consider the linear mapping $T:X\to X$ such that

$$Tg(x) :=\frac{\alpha}{2+\alpha} g \biggl(\frac{2+\alpha}{\alpha}x \biggr) $$

for all $x \in\mathbb{A}$ and for all $g \in X$. Letting $\mu=1$ and $y=x$ in (3.3), we get

$$ \biggl\Vert h \biggl( \frac{2+\alpha}{\alpha} x \biggr) - \frac{2+\alpha }{\alpha} h(x) \biggr\Vert _{\mathbb{A}} \leq\phi(x,x,x) $$

for all $x\in\mathbb{A}$, so we have

$$ \biggl\Vert h(x) - \frac{\alpha}{2+\alpha}h \biggl( \frac{2+\alpha}{\alpha} x \biggr) \biggr\Vert _{\mathbb{A}} \leq\frac{\alpha}{2+\alpha}\phi(x,x,x) $$

for all $x\in\mathbb{A}$. Hence, we obtain

$$d(h,Th) \leq\frac{\alpha}{2+\alpha}. $$

It follows from the proof of Theorem 2.1 that

$$ d(f,Tf) \leq\frac{1}{2+\alpha}. $$

By the same reasoning as the proof of Theorem 2.1, there exist a unique involutive $\mathbb{C}$-linear mapping $\delta: \mathbb {A} \to\mathbb{A}$ and a mapping $\theta: \mathbb{A} \to\mathbb{A}$ satisfying (3.5) and (3.6), respectively. The mappings *δ* and *θ* are given by

$$ \delta(x)=\lim_{n\to\infty} \biggl( \frac{\alpha}{2+\alpha} \biggr)^{n} f \biggl( \biggl(\frac{2+\alpha}{\alpha} \biggr)^{n} x \biggr) $$

and

$$ \theta(x)=\lim_{n\to\infty} \biggl( \frac{\alpha}{2+\alpha} \biggr)^{n} h \biggl( \biggl(\frac{2+\alpha}{\alpha} \biggr)^{n} x \biggr) $$

for all $x \in\mathbb{A}$, respectively. It follows from (3.2) that

$$\begin{aligned} & \bigl\Vert \delta(xyz) -\delta(xy)\theta(z) + \theta(x)\delta(y)\theta (z) - \theta(x)\delta(yz) \bigr\Vert _{\mathbb{A}} \\ &\quad = \biggl\Vert \lim_{n\to\infty} \biggl( \frac{\alpha}{2+\alpha} \biggr)^{3n} f \biggl( \biggl(\frac{2+\alpha}{\alpha} \biggr)^{3n} xyz \biggr) \\ &\qquad{}- \lim_{n\to\infty} \biggl( \frac{\alpha}{2+\alpha} \biggr)^{2n} f \biggl( \biggl(\frac{2+\alpha}{\alpha} \biggr)^{2n} xy \biggr) \cdotp\lim _{n\to \infty} \biggl( \frac{\alpha}{2+\alpha} \biggr)^{n} h \biggl( \biggl(\frac {2+\alpha}{\alpha} \biggr)^{n} z \biggr) \\ &\qquad{}+ \lim_{n\to\infty} \biggl( \frac{\alpha}{2+\alpha} \biggr)^{n} h \biggl( \biggl(\frac{2+\alpha}{\alpha} \biggr)^{n} x \biggr) \cdotp\lim _{n\to\infty} \biggl( \frac{\alpha}{2+\alpha} \biggr)^{n} f \biggl( \biggl(\frac{2+\alpha}{\alpha} \biggr)^{n} y \biggr) \\ &\qquad {}\cdotp\lim_{n\to\infty} \biggl( \frac{\alpha}{2+\alpha} \biggr)^{n} h \biggl( \biggl(\frac{2+\alpha}{\alpha} \biggr)^{n} z \biggr) \\ &\qquad{}- \lim_{n\to\infty} \biggl( \frac{\alpha}{2+\alpha} \biggr)^{n} h \biggl( \biggl(\frac{2+\alpha}{\alpha} \biggr)^{n} x \biggr) \cdotp\lim_{n\to\infty} \biggl( \frac{\alpha}{2+\alpha} \biggr)^{2n} f \biggl( \biggl(\frac{2+\alpha}{\alpha} \biggr)^{2n} yz \biggr) \biggr\Vert _{\mathbb{A}} \\ &\quad = \lim_{n\to\infty} \biggl( \frac{\alpha}{2+\alpha} \biggr)^{3n} \biggl\Vert f \biggl( \biggl(\frac{2+\alpha}{\alpha} \biggr)^{3n} xyz \biggr) -f \biggl( \biggl(\frac{2+\alpha}{\alpha} \biggr)^{2n} xy \biggr) \cdotp h \biggl( \biggl(\frac{2+\alpha}{\alpha} \biggr)^{n} z \biggr) \\ &\qquad{}+ h \biggl( \biggl(\frac{2+\alpha}{\alpha} \biggr)^{n} x \biggr) \cdotp f \biggl( \biggl(\frac{2+\alpha}{\alpha} \biggr)^{n} y \biggr) \cdotp h \biggl( \biggl(\frac{2+\alpha}{\alpha} \biggr)^{n} z \biggr) - h \biggl( \biggl( \frac{2+\alpha}{\alpha} \biggr)^{n} x \biggr) \\ & \qquad {}\cdotp f \biggl( \biggl(\frac{2+\alpha}{\alpha} \biggr)^{2n} yz \biggr) \biggr\Vert _{\mathbb{A}} \\ &\quad \leq \lim_{n\to\infty} \biggl( \frac{\alpha}{2+\alpha} \biggr)^{3n} \phi \biggl( \biggl(\frac{2+\alpha}{\alpha} \biggr)^{n} x, \biggl(\frac {2+\alpha}{\alpha} \biggr)^{n} y, \biggl(\frac{2+\alpha}{\alpha} \biggr)^{n} z \biggr) \\ &\quad \leq \lim_{n\to\infty} \biggl( \frac{\alpha}{2+\alpha} \biggr)^{n} \phi \biggl( \biggl(\frac{2+\alpha}{\alpha} \biggr)^{n} x, \biggl(\frac {2+\alpha}{\alpha} \biggr)^{n} y, \biggl(\frac{2+\alpha}{\alpha} \biggr)^{n} z \biggr) = 0 \end{aligned}$$

for all $x,y,z\in\mathbb{A}$. Hence

$$\delta(xyz) = \delta(xy)\theta(z) - \theta(x)\delta(y)\theta(z) + \theta (x) \delta(yz) $$

for all $x,y,z \in\mathbb{A}$. Next, we can show that $\theta: \mathbb {A} \to\mathbb{A}$ is $\mathbb{C}$-linear. Firstly, we will show that, for any $x \in\mathbb{A}$, $\mu(\theta x) = \theta(\mu x)$ for all $\mu\in\mathbb{S}$. For each $\mu\in\mathbb{S}$, substituting $x,y,z$ in (3.3) by $( \frac{2+\alpha}{\alpha} )^{n}x$, we obtain

3.7 $$\begin{aligned} & \biggl\Vert \mu h \biggl( \biggl( \frac{2+\alpha}{\alpha} \biggr)^{n+1} x \biggr) - \frac{2+\alpha}{\alpha} h \biggl(\mu \biggl( \frac{2+\alpha}{\alpha} \biggr)^{n} x \biggr) \biggr\Vert _{\mathbb{A}} \\ &\quad \leq\phi \biggl( \biggl( \frac{2+\alpha}{\alpha} \biggr)^{n} x, \biggl( \frac {2+\alpha}{\alpha} \biggr)^{n} x, \biggl( \frac{2+\alpha}{\alpha} \biggr)^{n} x \biggr) \end{aligned}$$

for all $x \in\mathbb{A}$. For $\mu= 1$, we also have

3.8 $$ \begin{aligned}[b] & \biggl\Vert h \biggl( \biggl( \frac{2+\alpha}{\alpha} \biggr)^{n+1} x \biggr) - \frac{2+\alpha}{\alpha} h \biggl( \biggl( \frac{2+\alpha}{\alpha} \biggr)^{n} x \biggr) \biggr\Vert _{\mathbb{A}} \\ &\quad \leq\phi \biggl( \biggl( \frac{2+\alpha}{\alpha} \biggr)^{n} x, \biggl( \frac {2+\alpha}{\alpha} \biggr)^{n} x, \biggl( \frac{2+\alpha}{\alpha} \biggr)^{n} x \biggr) \end{aligned} $$

for all $x \in\mathbb{A}$. It follows from (3.7) and (3.8) that

$$\begin{aligned} & \biggl\Vert \frac{2+\alpha}{\alpha} h \biggl(\mu \biggl( \frac{2+\alpha}{\alpha} \biggr)^{n} x \biggr) - \frac{2+\alpha}{\alpha} \mu h \biggl( \biggl( \frac{2+\alpha }{\alpha} \biggr)^{n} x \biggr) \biggr\Vert _{\mathbb{A}} \\ &\quad = \biggl\Vert \frac{2+\alpha}{\alpha} h \biggl(\mu \biggl( \frac{2+\alpha }{\alpha} \biggr)^{n} x \biggr) - \mu h \biggl( \biggl( \frac{2+\alpha}{\alpha} \biggr)^{n+1} x \biggr) \\ &\qquad{}+ \mu h \biggl( \biggl( \frac{2+\alpha}{\alpha} \biggr)^{n+1} x \biggr) - \frac{2+\alpha}{\alpha} \mu h \biggl( \biggl( \frac{2+\alpha }{\alpha} \biggr)^{n} x \biggr) \biggr\Vert _{\mathbb{A}} \\ &\quad \leq \biggl\Vert \frac{2+\alpha}{\alpha} h \biggl(\mu \biggl( \frac{2+\alpha }{\alpha} \biggr)^{n} x \biggr) - \mu h \biggl( \biggl( \frac{2+\alpha}{\alpha} \biggr)^{n+1} x \biggr) \biggr\Vert _{ \mathbb{A}} \\ &\qquad{}+ \biggl\Vert \mu h \biggl( \biggl( \frac{2+\alpha}{\alpha} \biggr)^{n+1} x \biggr) - \frac{2+\alpha}{\alpha} \mu h \biggl( \biggl( \frac{2+\alpha }{\alpha} \biggr)^{n} x \biggr) \biggr\Vert _{\mathbb{A}} \\ &\quad = \biggl\Vert \frac{2+\alpha}{\alpha} h \biggl(\mu \biggl( \frac{2+\alpha }{\alpha} \biggr)^{n} x \biggr) - \mu h \biggl( \biggl( \frac{2+\alpha}{\alpha} \biggr)^{n+1} x \biggr) \biggr\Vert _{ \mathbb{A}} \\ &\qquad{}+ \vert \mu \vert \biggl\Vert h \biggl( \biggl( \frac{2+\alpha}{\alpha} \biggr)^{n+1} x \biggr) - \frac{2+\alpha}{\alpha} h \biggl( \biggl( \frac{2+\alpha}{\alpha } \biggr)^{n} x \biggr) \biggr\Vert _{\mathbb{A}} \\ &\quad \leq 2\phi \biggl( \biggl(\frac{2+\alpha}{\alpha} \biggr)^{n} x, \biggl( \frac{2+\alpha}{\alpha} \biggr)^{n} x, \biggl(\frac{2+\alpha}{\alpha} \biggr)^{n} x \biggr) \end{aligned}$$

for all $x \in\mathbb{A}$. This implies that

$$\begin{aligned} & \biggl\Vert \biggl(\frac{\alpha}{2+\alpha} \biggr)^{n} h \biggl( \biggl( \frac {2+\alpha}{\alpha} \biggr)^{n} \mu x \biggr) - \biggl( \frac{\alpha}{2+\alpha } \biggr)^{n} \mu h \biggl( \biggl( \frac{2+\alpha}{\alpha} \biggr)^{n} x \biggr) \biggr\Vert _{\mathbb{A}} \\ &\quad \leq \frac{2\alpha}{2+\alpha} \biggl(\frac{\alpha}{2+\alpha} \biggr)^{n} \phi \biggl( \biggl(\frac{2+\alpha}{\alpha} \biggr)^{n} x, \biggl( \frac {2+\alpha}{\alpha} \biggr)^{n} x, \biggl(\frac{2+\alpha}{\alpha} \biggr)^{n} x \biggr) \end{aligned}$$

for all $x \in\mathbb{A}$. By (2.2), we have

$$ \lim_{n \to\infty} \biggl\Vert \biggl(\frac{\alpha}{2+\alpha} \biggr)^{n} h \biggl( \biggl( \frac{2+\alpha}{\alpha} \biggr)^{n} \mu x \biggr) - \biggl(\frac{\alpha}{2+\alpha} \biggr)^{n} \mu h \biggl( \biggl( \frac{2+\alpha }{\alpha} \biggr)^{n} x \biggr) \biggr\Vert _{\mathbb{A}} = 0 $$

for all $x \in\mathbb{A}$. That is,

$$ \theta(\mu x) = \mu\theta( x) $$

for all $x \in\mathbb{A}$. By Lemma 1.5, we obtain that *θ* is a $\mathbb{C}$-linear mapping. Thus, $\delta: \mathbb{A} \to\mathbb {A}$ is generalized *θ*-derivation satisfying (3.5). □

### Corollary 3.2

*Let* $p \in[0,1)$, $\varepsilon\in[0,\infty)$ *and* *f* *be a mapping of* $\mathbb{A}$ *into itself such that*

3.9 $$\begin{aligned}& \bigl\Vert E_{\mu} f(x,y,z) \bigr\Vert _{\mathbb{A}} \leq \varepsilon\bigl( \Vert x \Vert _{\mathbb{A}}^{p}+ \Vert y \Vert _{\mathbb{A}}^{p}+ \Vert z \Vert _{\mathbb{A}}^{p}\bigr), \end{aligned}$$

3.10 $$\begin{aligned}& \bigl\Vert f(xyz) - f(xy)\theta(z) + \theta(x)f(y)\theta(z) - \theta(x)f(yz) \bigr\Vert _{\mathbb{A}} \leq \varepsilon\bigl( \Vert x \Vert _{\mathbb{A}}^{p}+ \Vert y \Vert _{\mathbb {A}}^{p}+ \Vert z \Vert _{\mathbb{A}}^{p}\bigr), \end{aligned}$$

3.11 $$\begin{aligned}& \biggl\Vert \mu h \biggl( \frac{2+\alpha}{2\alpha}(x+y) \biggr) - \frac {2+\alpha}{2\alpha} \bigl(h(\mu x) + h(\mu y) \bigr) \biggr\Vert _{\mathbb {A}} \leq \varepsilon\bigl( \Vert x \Vert _{\mathbb{A}}^{p}+ \Vert y \Vert _{\mathbb{A}}^{p} + \Vert x \Vert _{\mathbb{A}}^{p} \bigr), \end{aligned}$$

3.12 $$\begin{aligned}& \bigl\Vert f\bigl(x^{*}\bigr) - f(x)^{*} \bigr\Vert _{\mathbb{A}} \leq3\varepsilon \Vert x \Vert ^{r}_{\mathbb{A}} \end{aligned}$$

*for all* $\mu\in\mathbb{S}$ *and for all* $x,y,z \in\mathbb{A}$. *Then there exist unique* $\mathbb{C}$-*linear mappings* $\delta,\theta: \mathbb {A} \to\mathbb{A}$ *such that*

$$\begin{aligned}& \bigl\Vert f(x) - \delta(x) \bigr\Vert _{\mathbb{A}} \leq \frac{3\varepsilon}{ (1- ( \frac{2+\alpha}{\alpha} )^{p-1} )(2+\alpha)} \Vert x \Vert ^{p}_{\mathbb{A}}, \\& \bigl\Vert h(x) - \theta(x) \bigr\Vert _{\mathbb{A}} \leq \frac{\varepsilon\alpha}{ (1- ( \frac{2+\alpha}{\alpha} )^{p-1} )(2+\alpha)} \Vert x \Vert ^{p}_{\mathbb{A}}, \end{aligned}$$

*for all* $x \in\mathbb{A}$. *Moreover*, $\delta: \mathbb{A} \to\mathbb {A}$ *is a generalized* *θ*-*derivation on* $\mathbb{A}$.

### Proof

The proof follows from Theorem 3.1 by taking

$$\phi(x,y,z) = \varepsilon\bigl( \Vert x \Vert _{\mathbb{A}}^{p}+ \Vert y \Vert _{\mathbb{A}}^{p}+ \Vert z \Vert _{\mathbb{A}}^{p}\bigr) $$

for all $x,y,z \in\mathbb{A}$. Then $k= ( \frac{2+\alpha}{\alpha} )^{p-1}$ and we get the desired results. □

### Theorem 3.3

*Let* $\phi: \mathbb{A}^{3} \to[0,\infty)$ *such that there exists a* $k<1$ *satisfying*

$$ \phi(x,y,z) \leq \biggl(\frac{\alpha}{2+\alpha} \biggr)^{3} k \phi \biggl( \frac{2+\alpha}{\alpha}x, \frac{2+\alpha}{\alpha}y, \frac{2+\alpha }{\alpha}z \biggr) $$

*for all* $x,y,z \in\mathbb{A}$. *Let* $f, h$ *be mappings of* $\mathbb{A}$ *into itself satisfying* (3.1), (3.2), (3.3) *and* (3.4). *Then there exist unique* $\mathbb {C}$-*linear mappings* $\delta,\theta: \mathbb{A} \to\mathbb{A}$ *such that*

$$\begin{aligned}& \bigl\Vert f(x) - \delta(x) \bigr\Vert _{\mathbb{A}} \leq \frac{\alpha^{2} k}{(1-k)(2+\alpha )^{3}}\phi(x,x,x), \\& \bigl\Vert h(x) - \theta(x) \bigr\Vert _{\mathbb{A}} \leq \frac{ k}{1-k} \biggl(\frac {\alpha}{2+\alpha} \biggr)^{3}\phi(x,x,x) \end{aligned}$$

*for all* $x \in\mathbb{A}$. *Moreover*, $\delta: \mathbb{A} \to\mathbb {A}$ *is a generalized* *θ*-*derivation on* $\mathbb{A}$.

### Proof

The proof is similar to the proofs of Theorem 2.3 and Theorem 3.1. □

### Corollary 3.4

*Let* $p \in(3,\infty]$, $\varepsilon\in[0,\infty)$ *and* *f* *be a mapping of* $\mathbb{A}$ *into itself satisfying* (3.9), (3.10), (3.11) *and* (3.12). *Then there exist unique* $\mathbb{C}$-*linear mappings* $\delta,\theta: \mathbb{A} \to\mathbb{A}$ *such that*

$$\begin{aligned}& \bigl\Vert f(x) - \delta(x) \bigr\Vert _{\mathbb{A}} \leq \frac{3\alpha^{2}\varepsilon }{ ( ( \frac{2+\alpha}{\alpha} )^{p-3}-1 )(2+\alpha )^{3}} \Vert x \Vert ^{p}_{\mathbb{A}}, \\& \bigl\Vert h(x) - \theta(x) \bigr\Vert _{\mathbb{A}} \leq \frac{\varepsilon}{ ( \frac {2+\alpha}{\alpha} )^{p-3}-1}\cdotp \biggl(\frac{\alpha}{2+\alpha } \biggr)^{3} \Vert x \Vert ^{p}_{\mathbb{A}} \end{aligned}$$

*for all* $x \in\mathbb{A}$. *Moreover*, $\delta: \mathbb{A} \to\mathbb {A}$ *is a generalized* *θ*-*derivation* $\mathbb{A}$.

### Proof

The proof follows from Theorem 3.3 by taking

$$\phi(x,y,z) = \varepsilon\bigl( \Vert x \Vert _{\mathbb{A}}^{p}+ \Vert y \Vert _{\mathbb{A}}^{p}+ \Vert z \Vert _{\mathbb{A}}^{p}\bigr) $$

for all $x,y,z \in\mathbb{A}$. Then $k= ( \frac{\alpha}{2+\alpha} )^{p-3}$ and we get the desired results. □

We recall definition of generalized derivations on $C^{*}$-algebra.

### Definition 3.2

([13])

A generalized derivation $\delta: \mathbb{A} \to\mathbb{A}$ is involutive $\mathbb{C}$-linear and satisfies

$$ \delta(xyz) = \delta(xy)z - x\delta(y)z+ x\delta(yz) $$

for all $x,y,z \in\mathbb{A}$.

### Remark 3.5

According to Definition 3.1, If $\theta= I$, *I* is identity mapping on $\mathbb{A}$, then a generalized *θ*-derivation is a generalized derivation. If the mapping *h* is identity mapping and $\alpha= 2$, Then Theorem 3.1 and Theorem 3.3 we recover Theorem 3.2 and Theorem 3.4 in [10], respectively. Moreover, if we set the mapping *h* is identity mapping, $\alpha= 2$ and $\phi(x,y,z) = \varepsilon\cdotp\|x\|^{\frac{p}{3}}_{\mathbb{A}} \cdotp\|y\|^{\frac{p}{3}}_{\mathbb{A}}\cdotp\|z\|^{\frac{p}{3}}_{\mathbb{A}}$ in Theorem 3.1 where $p\in[0,1)$ and $\varepsilon\in [0,\infty)$, then Theorem 3.1 one recovers Corollary 3.3 in [10] with $k= ( \frac{2+\alpha}{\alpha} )^{p-1}$.

## Conclusions

In the first section of main results, we prove Hyers–Ulam–Rassias stability of $C^{*}$-algebra homomorphisms for the generalized Cauchy–Jensen equation $C^{*}$-algebras by using fixed point alternative theorem. In the second section of main results, we introduce and investigate the Hyers–Ulam–Rassias stability of generalized *θ*-derivation for such function $C^{*}$-algebras by the same method. By our main results we recover partial results of Park and An in [10] by Remark 2.5 and Remark 3.5.
